# A Luminol-Based, Peroxide-Free Fenton Chemiluminescence System Driven by Cu(I)-Polyethylenimine-Lipoic Acid Nanoflowers for Ultrasensitive SARS-CoV-2 Immunoassay

**DOI:** 10.3390/bios16010061

**Published:** 2026-01-14

**Authors:** Mahmoud El-Maghrabey, Ali Abdel-Hakim, Yuta Matsumoto, Rania El-Shaheny, Heba M. Hashem, Naotaka Kuroda, Naoya Kishikawa

**Affiliations:** 1 Department of Pharmaceutical Analytical Chemistry, Faculty of Pharmacy, Mansoura University, Mansoura 35516, Egypt; rania_yomna@mans.edu.eg (R.E.-S.); hebamaher89@mans.edu.eg (H.M.H.); 2 Department of Analytical Chemistry, Faculty of Pharmacy, University of Sadat City, Sadat City 32897, Monufia, Egypt; ali.hassan@fop.usc.edu.eg; 3 Department of Analytical Chemistry for Pharmaceuticals, Course of Pharmaceutical Sciences, Graduate School of Biomedical Sciences, Nagasaki University, 1-14 Bunkyo-machi, Nagasaki 852-8521, Japan; yuta.matsumoto1211@gmail.com (Y.M.); n-kuro@nagasaki-u.ac.jp (N.K.)

**Keywords:** nanoflower, peroxide-free immunoassay, lipoic acid, cuprous, Fenton’s reaction, chemiluminescence, SARS-CoV-2

## Abstract

The reliance on unstable hydrogen peroxide (H_2_O_2_) adversely affects the robustness and simplicity of chemiluminescence (CL)-based immunoassays. We report a novel external H_2_O_2_-free Fenton CL system integrated into a highly sensitive non-enzymatic immunoassay for the detection of SARS-CoV-2 nucleoprotein, utilizing cuprous–polyethylenimine–lipoic acid nanoflowers (Cu(I)-PEI-LA-Ab NF) as a non-enzymatic tag. The signaling polymer (PEI-LA) was synthesized via EDC/NHS coupling, which conjugated approximately 550 LA units to the PEI backbone. This polymer formed antibody-conjugated NF with various metal ions, and the Cu(I)-based variant was selected for its intense and sustained CL with luminol. The mechanism relies on an in situ Fenton reaction, in which dissolved oxygen is reduced by Cu(I) to H_2_O_2_, which reacts with oxidized Cu(II), producing hydroxyl radicals that oxidize luminol. Direct calibration of the SARS-CoV-2 nucleoprotein fixed on microplate wells demonstrated excellent linearity in the range of 0.01–3.13 ng/mL (LOD = 3 pg/mL). In a final competitive immunoassay format for samples spiked with the antigen, a decreasing CL signal that correlated with increasing antigen concentration was obtained in the range of 0.1–20.0 ng/mL, achieving excellent recoveries that were favorable compared with those of the sandwich ELISA kit, establishing this H_2_O_2_-independent platform as a powerful and robust tool for clinical diagnostics.

## 1. Introduction

The enzyme-linked immunosorbent assay (ELISA), also known as the enzyme immunoassay (EIA), is based on specific antigen–antibody interactions. This involves immobilizing the antigen or antibody on a solid microplate surface, followed by a signal readout via enzymatic or fluorogenic reactions. In the typical 96- or 384-well plate format, capture antibodies bind to their corresponding antigens, which are then detected using enzyme-conjugated secondary antibodies. Upon substrate addition, enzymes such as horseradish peroxidase (HRP) or alkaline phosphatase (ALP) catalyze reactions that produce colorimetric, fluorescence, or chemiluminescence (CL) signals that can be measured as optical density [1,2]. There are four major types of ELISA: direct, indirect, sandwich, and competitive ELISA. Each format varies according to the antigen immobilization and detection procedure. Direct ELISA uses an enzyme-labeled primary antibody for rapid detection, whereas indirect ELISA uses a secondary enzyme-linked antibody to amplify the signals. Sandwich ELISA, the most sensitive format, uses matched antibody pairs that bind to two distinct epitopes on the same antigen, enhancing specificity for complex samples [2,3,4,5]. Competitive ELISA measures the signal inhibition caused by analyte competition and is often used for small molecules and peptides, where dual epitope binding is not possible [3]. Because of its precision, scalability, and reproducibility, ELISA remains the diagnostic standard for infectious diseases, such as HIV [6], hepatitis [7], and SARS-CoV-2 [8,9].

Signal transduction in traditional ELISA depends on enzymatic catalysis and its coupling to optical events that can be quantified. Standard labeling enzymes include horseradish peroxidase (HRP) and alkaline phosphatase (ALP) because of their wide substrate selection, low cost, and robust catalytic turnover. HRP typically oxidizes luminol or tetramethylbenzidine (TMB) in the presence of hydrogen peroxide (H_2_O_2_), whereas ALP catalyzes phosphate removal to yield p-nitrophenol chromophores [3,10]. However, enzymatic reagents suffer from poor stability, temperature sensitivity, and susceptibility to inhibitors. Additionally, these enzymes depend on unstable H_2_O_2_ as an essential reagent, which affects the reliability and ease of enzymatic immunoassays.

Over the past five decades, ELISA has undergone significant evolution, from enzyme-based formats to nanomaterial-driven and non-enzymatic CL adaptations, which have overcome the stability, sensitivity, and operational limitations of classical ELISA systems. Synthetic and nanoparticle-based alternatives have emerged as next-generation immunoassay tags for these applications. Gold nanoparticles (AuNPs) [11], quantum dots (QDs) [12], and carbon nanomaterials [13] exhibit high extinction coefficients, quantum yields, and superior photostability. For instance, AuNP-based immunoassays enable colorimetric changes that are detectable by the naked eye through aggregation-induced plasmonic shifts. In contrast, QD-labeled assays use tunable fluorescence to provide multiplexed and quantitative readouts. Despite these improvements, reproducibility and stability remain challenges, prompting a shift toward enzyme-free and artificial catalysis-driven immunoassays [14].

Non-enzymatic immunoassays (NEIAs) represent a critical evolution in immunodiagnostics, replacing fragile biological enzymes with robust synthetic or inorganic catalysts. These assays often rely on nanozymes, redox-active complexes, or hybrid nanostructures that mimic HRP or ALP activity but do not degrade under harsh storage or reaction conditions. Recent examples include the quinone-linked immunosorbent assay (QuLISA) [2,15,16], polymerized alizarin red–inorganic hybrid nanoarchitecture-linked immunosorbent assay (PARIHN-LISA) [4], and nanoflower-linked immunosorbent assay (NF-LISA) [17]. QuLISA integrates quinone-functionalized molecules as redox labels, which are capable of direct electron transfer [18]. In PARIHN-LISA, a chitosan–alizarin polymer hybridized with zinc ions forms a stable fluorescent labeling system for SARS-CoV-2 nucleoprotein (NP) detection, exploiting alizarin’s boric acid-induced fluorescence with detection limits down to the picomolar level. These enzyme-free strategies demonstrate high sensitivity, rapid response, and sustainability, addressing the issues of enzyme instability, high cost, and complex storage requirements of enzyme-based sensors.

Organic–inorganic hybrid nanoflowers (HNFs or NFs) are hierarchically structured materials formed by the self-assembly of metal ions with organic biomolecules such as enzymes, DNA, or polyamines. Their petal-like morphology endows them with exceptionally high surface area, porosity, and active site density, which are ideal for immobilizing biomolecules and catalyzing reactions with enhanced efficiency [17,19,20,21]. The synthesis process typically involves coordination between transition metal ions (Cu(II), Co(II), Zn(II), etc.) and organic ligands, forming crystalline–amorphous hybrid architectures. These NFs exhibit improved catalytic activity, storage stability, and reusability compared to free enzymes, making them excellent supports for biosensors, bioassays, and environmental monitoring. In particular, HNFs incorporating Cu(II) possess strong redox properties that can sustain catalytic cycles similar to those of natural peroxidases and oxidases [5,17], which is key to the development of peroxide-free CL systems.

NF-LISA builds upon the hybrid NF concept by embedding antibodies and catalytic metals within an inorganic–organic hybrid matrix to generate non-enzymatic signal tags. These NF-LISAs exhibit remarkable signal amplification owing to the dense loading of catalytically active sites and bioligands on the petal-like surfaces [17,19]. This approach allows the direct labeling of the detection antibody with the labeling tag without using the troublesome avidin–biotin system, which suffers from the non-selective binding of avidin to matrix bio-contents [22]. For example, Yin et al. created antibody-functionalized enzymatic Cu(II) phosphate NFs and used them in ELISA to detect Staphylococcus aureus. The ELISA relies on a TMB-H_2_O_2_ system to amplify the signal [20]. To further enhance the efficacy of target recognition, Su et al. developed protein-inorganic hybrid ss by encasing HRP inside a phosphate-crystal scaffold [21]. While NF-based ELISAs that rely on enzymes show promise for future diagnostic uses, enzymes are expensive, have low stability, and lose their activity rapidly in relatively hostile environments [16,23]. Although enzymes are more stable when incorporated into NFs than in their free forms, they still degrade with time. Manufacturing expenses are increasing because of the need for suitable storage conditions and additional processes to load the enzyme into the NFs. Additionally, these approaches require the presence of H_2_O_2_ in the system for the reaction to occur and for a discernible signal to be generated. Recently, chitosan–alizarin and Cu(II)-based NFs were developed by our research group, which demonstrated dual catalytic and binding capacities, combining metal-centered redox activity with bio-affinity recognition for target analytes. Compared with conventional ELISA formats, our developed NF-LISA systems do not require H_2_O_2_ substrates or enzyme stabilization buffers, enabling a longer shelf life, higher robustness, and simplified procedures than conventional ELISA formats. Their intrinsic catalytic activity directly drives chromogenic or CL reactions, offering picogram-to-femtogram-level sensitivity for biological detection [5].

In this study, we continued our innovative work in developing a peroxide-free ultrasensitive CL NF-LISA, introducing a novel luminol-based, external peroxide-free Fenton CL system mediated by cuprous–polyethylenimine–lipoic acid NFs (Cu(I)–PEI–LA NFs) for ultrasensitive SARS-CoV-2 immunoassays. Unlike conventional HRP–luminol systems that depend on unstable H_2_O_2_ as an oxidant, this platform exploits an in situ Fenton-like reaction in which Cu(I) directly reduces the dissolved oxygen to generate hydroxyl radicals that activate luminol oxidation in the absence of exogenous peroxide. This results in a highly stable and sustainable CL mechanism with over 100-fold signal enhancement compared to traditional Cu(II) and Fe(II) analogs. The polycationic polymer PEI serves as both a structural scaffold and electronic mediator, while LA provides disulfide anchoring and redox-cyclic functionality, yielding an architecture capable of cycling Cu(I)/Cu(II) species efficiently. The resulting Cu(I)–PEI–LA–antibody (AB) NF tag combines catalytic robustness, high luminescence output, and superior antigen-binding capacity in a single nanostructure. The elimination of peroxides not only stabilizes the system but also removes the decomposition variability intrinsic to enzyme-based HRP/H_2_O_2_ systems. This work presents a new Cu(I)-based, H_2_O_2_-independent CL immunosensor through a first-of-its-kind external hydrogen peroxide-free Fenton’s reaction. Our previously reported Cu(II)–chitosan–alizarin NF label also operated without external H_2_O_2_, establishing the first generation of H_2_O_2_-free CL immunosensors [5]. The present Cu(I)–PEI–LA–luminol platform constitutes a second-generation design with a different redox backbone and superior CL output and sensitivity, achieving over 100-fold signal enhancement and markedly improved sensitivity compared with our previous Cu(II)–chitosan–alizarin NF system and other reported labels, establishing a paradigm shift toward more robust, stable, and sustainable diagnostic platforms.

## 2. Materials and Methods

### 2.1. Chemicals and Reagents

Lipoic acid (LA), dimethyl formamide (DMF), 1-ethyl-3-(3-dimethylaminopropyl)carbodiimide (EDC), N-hydroxysuccinimide (NHS), NaOH, copper(I) thiocyanate (CuSCN), CoCl_2_, MnO_2_, CdCl_2_, and 10,10′-dimethyl-9,9′-biacridinium dinitrate (lucigenin) were purchased from Tokyo Chemical Industry (Tokyo, Japan). Anti-SARS-CoV-2 nucleocapsid and COVID-19 nucleocapsid proteins were obtained from Cosmo Bio. Ltd. (Tokyo, Japan) and Abeomics INC. (San Diego, CA, USA). A sandwich ELISA kit for the detection of SARS-CoV-2 NP was obtained from LifeSpan BioSciences (San Diego, CA, USA). Polyethylenimine (PEI, average Mw ~25,000), FeCl_2_, superoxide dismutase (SOD), mannitol, and catalase were purchased from Sigma-Aldrich (St. Louis, MO, USA). Luminol, sodium chloride, and disodium hydrogen phosphate were obtained from Nacalai-Tesque (Kyoto, Japan). Potassium chloride, sodium azide, CuSO_4_, and potassium dihydrogen phosphate were obtained from Wako Pure Chemical Industries, Ltd. (Osaka, Japan). Distilled water was prepared using a Yamato Autostill WG203 device (Tokyo, Japan). Phosphate-buffered saline (PBS; 100 mM, pH 7.4) was prepared by mixing sodium chloride, potassium chloride, potassium dihydrogen phosphate, and disodium hydrogen phosphate in the appropriate proportions.

### 2.2. Instruments

Microplate CL measurements were performed using a SpectraMax M5 and SpectraMax L microplate reader (Molecular Devices, San Jose, CA, USA) operated with SoftMax Pro 5 software (Molecular Devices, San Jose, CA, USA) using polystyrene, white, transparent, and F-bottom 96-well cell culture microplates (CELLSTAR^®^, T, L.I.D., sterile, Greiner Bio-One Co., Ltd., Tokyo, Japan). A pH meter (F-71, Horiba, Kyoto, Japan) was used to measure pH. Characterization tests were performed using a JEOL scanning electron microscope (SEM) model JSM-7500F (Tokyo, Japan) operated at a voltage of 15 kV and K-Alpha X-ray photoelectron spectroscopy (XPS) (Waltham, MA, USA). Attenuated complete reflection Fourier-transform infrared (ATR-FTIR) spectra were obtained on a Bruker ALPHA II spectrometer (Bruker, Rosenheim, Germany). X-ray diffraction (XRD) observations were performed using a Rigaku SmartLab SE diffractometer (Rigaku, Tokyo, Japan) with an accelerating voltage of 40 kV and a tube current of 50 mA. The equipment included a Cu Kβ filter and a one-dimensional detector and was operated using the manufacturer’s software. Diffraction data were gathered over a 2θ range of 5–80°, with a step size of 0.01° and a scan speed of 50° per minute.

### 2.3. Synthesis of Different NFs of PEI Polymerized LA (PEI-LA)-SARS-CoV-2 NP Ab Using Six Metals

LA polymer was synthesized using PEI as the polymer backbone. In 20 mL DMF, 3.0 mM of LA, 3.0 mM of EDC, and 1.6 mM of NHS were mixed and left to react for 1.0 h under N_2_ purging for activation of the carboxylic group of LA. Subsequently, 1.0 μM PEI in 10 mL of DMF was added. The reaction mixture was then stirred for 24 h at room temperature under N_2_ purge. Finally, the product was filtered under vacuum, yielding white crystals of PEI-LA (yield: 50.3%).

Subsequently, a stock solution of PEI-LA (0.3 mg/mL) in 100 mM NaOH (pH 7.4) was prepared. Then, 100 µL of 1.0 mg/mL metal ion aqueous solution was added to 1 mL of the stock solution of PEI-LA, mixed, and incubated for 12 h at 4 °C. Six different metal ions were used to synthesize the NFs. The metal ions used were Cu(I):CuSCN, Cu(II):CuSO_4_, Fe(II): FeCl_2_, Co(II):CoCl_2_, Mn(IV):MnO_4_, and Cd(II):CdCl_2_. Subsequently, 100 µL of 1 µM SARS-CoV-2 NP Ab in PBS (pH 7.4) was added to the mixture and incubated for 24 h at 4 °C. Next, the solutions were centrifuged at 4000 rpm for five minutes and washed three times with deionized water. Then, the obtained NFs, namely “Cu(I)-PEI-LA-Ab, Cu(II)-PEI-LA-Ab, Fe(II)-PEI-LA-Ab, Co(II)-PEI-LA-Ab, Mn(IV)-PEI-LA-Ab, and Cd(II)-PEI-LA-Ab,” were diluted in PBS and stored in the refrigerator till further use.

### 2.4. CL Measurements of Different NFs and Different Components of the NFs

For measuring the CL intensity of the synthesized NFs, 50 µL of metals-PEI-LA-Ab was transferred to microplate wells; then, 50 µL of 400 µM lucigenin was dissolved in 100 mM carbonate buffer (pH 10), or 50 µL of 400 μM luminol in 40 mM NaOH was injected into the wells. The CL intensity was measured for 5 min for each CL reagent. The integrated CL intensity was calculated and plotted against the NF metal type to select the best one for the immunoassay of SARS-CoV-2 NP Ag.

To measure the CL intensity of the NF skeleton, 50 µL of the different components of the NFs, prepared alone or in combination, were added to 50 µL of 400 μM luminol in 40 mM NaOH. The CL intensity was measured for 5 min. The integrated CL intensity was calculated and plotted against the NF skeleton component.

### 2.5. CL Measurement Procedure of Fixed SARS-CoV-2 NP

One hundred microliters of 0.1 M PBS (pH 7.4) containing SARS-CoV-2 nucleocapsid protein at concentrations ranging from 0.01 to 3.13 ng/mL was added to a high-affinity 96-well microplate and incubated at 4 °C for 24 h to immobilize the antigen protein in the plate wells. To block the free sites in the wells, 0.3 mL of bovine serum albumin (BSA) at a concentration of 1% was applied and incubated at room temperature for a duration of two hours. After washing with PBS to remove unbound antigen proteins and excess BSA, 100 μL of Cu(I)-PEI-LA-Ab NFs dispersed in PBS (pH 7.4) was added and incubated at 37 °C for 2.0 h to form the immune complex. After another wash to remove unbound Cu(I)-PEI-LA-Ab NFs, the plate was placed in a plate reader, and 50 μL of 40 mM NaOH solution containing 450 μM luminol was added to measure the resulting CL for 300 s at 37 °C.

### 2.6. Procedure for the CL Competitive Immunoassay for the Determination of SARS-CoV-2 NP

One hundred microliters of 200 ng/mL SARS-CoV-2 NP in PBS (pH 7.4) was added to a microplate and incubated at 4 °C for 24 h. To block the free sites of the wells, 0.3 mL of BSA (1%) was applied and incubated at room temperature for two hours. After washing with PBS, 100 μL of Cu(I)-PEI-LA-Ab NFs dispersed in PBS (pH 7.4) and 100 μL of SARS-CoV-2 NP in PBS (pH 7.4) were added and incubated at 37 °C for 2.0 h. After another wash with PBS, the plate was set up in the plate reader, and 50 μL of 40 mM NaOH solution containing 450 μM luminol was added to measure the resulting CL for 300 s at 37 °C.

## 3. Results and Discussion

Our target was to develop a new non-enzymatic organic–inorganic hybrid NFs at room temperature for integration into a highly sensitive non-enzymatic immunoassay for SARS-CoV-2 NP detection. To achieve extremely sensitive detection of SARS-CoV-2 NP, we devised a new immunoassay that uses PEI as a backbone and LA as a disulfide source, which, when combined with a metal ion, can induce a Fenton-like CL reaction.

### 3.1. Synthesis and Investigation of the PEI-LA Polymer

**Figure 1:**
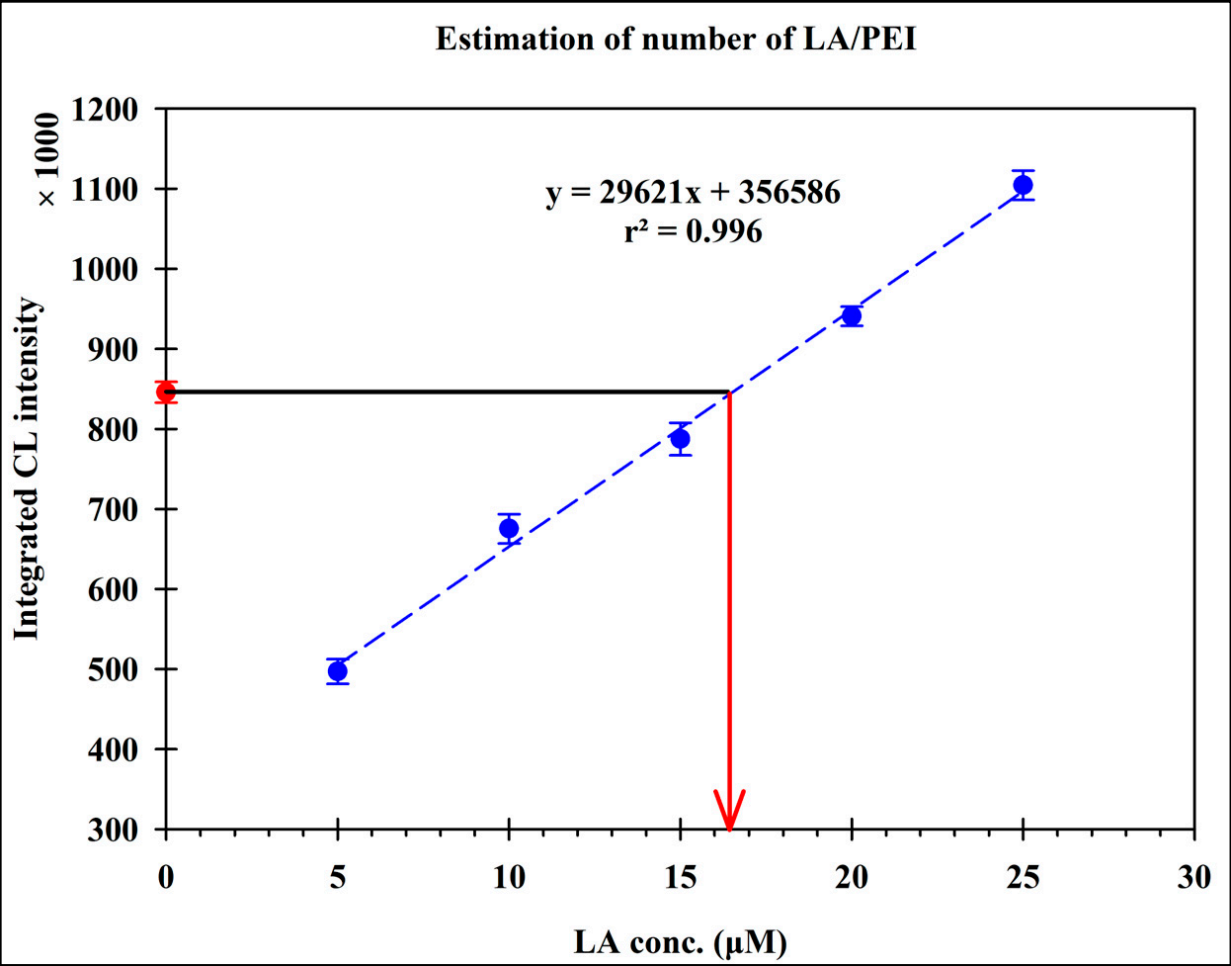
The number of LA molecules per PEI molecule was determined using the CL generation redox cycle reaction of LA with menadione and luminol, as described by Kishikawa et al. [24]. The linear curve shows the calibration curve of LA, and the arrows show the CL response of 0.03 μM PEI-LA.

PEI has 580 repeated units of ethyleneimine groups that can react with the carboxylic group of LA after EDC/NHS activation to form amide bonds. Next, it was essential to confirm the amount of LA in the synthesized PEI-LA. PEI-LA (0.03 μM) was reacted with menadione and luminol according to the CL method described by Kishikawa et al. to measure LA [24]. The formed CL was measured and compared with a calibration curve for LA (5.0–25.0 μM) using the same method. It was found that 0.03 μM PEI-LA gives a CL response equivalent to 16.5 μM (Figure 1), which means that one molecule of PEI is conjugated to approximately 550 LA units.

### 3.2. Optimization for the Synthesis of the NFs

After synthesizing the PEI-LA-functionalized polymer, we decided to improve the sensitivity and simplicity of Ab labeling by directly attaching the SARS-CoV-2 NP detection Ab to PEI-LA using less toxic and less complicated methods than the conventional methods of labeling Ab that either use several catalysts or an avidin–biotin system, adopting the NF synthesis approach at room temperature to be integrated into a highly sensitive non-enzymatic immunoassay for the detection of the SARS-CoV-2 NP. NFs are organic–inorganic hybrid architectures formed from a core metal, an anion counterpart, and an organic polymer. By directly attaching the detection Ab to PEI-LA via NF technology, we aimed to enhance the sensitivity and ease of antibody labeling. The primary component of NFs is a metal, which may be of many types, such as copper, iron, manganese, cobalt, and nickel. Structural and functional components can be found in organic or protein-based materials. Phosphate ions are often used as an anion counterpart to stabilize NFs and avoid agglomeration. Proteins and metal nanoparticles may be more easily attached, and the structure can be made more stable with the aid of a polymer component.

**Figure 2:**
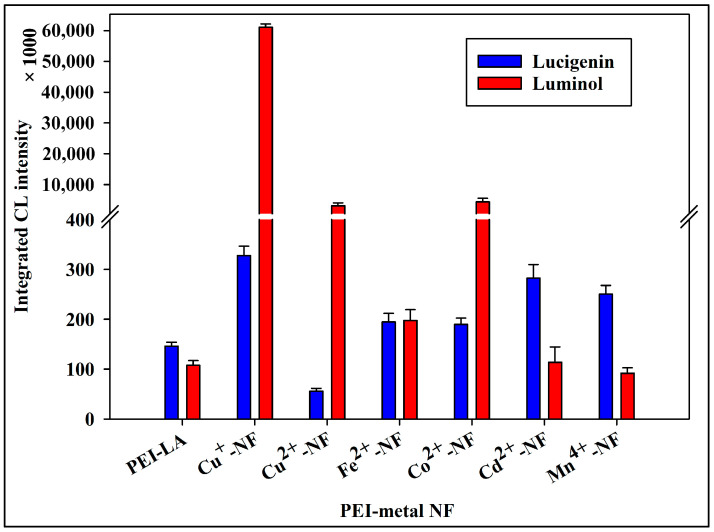
CL performance of the different synthesized SARS-CoV-2 NP Ab PEI-metal NFs using lucigenin and luminol as CL reagents.

In our approach, Ab was the protein component of our proposed NF method, and a PEI-LA functionalized polymer was the polymer responsible for signal production in conjunction with the metal. At first, NFs were synthesized using PEI-LA and different metal ions. The selection of the metal was based on its unique ability, which plays a crucial role in its application. Six different metal ions were tested for the synthesis of Ab-labeled PEI-LA NFs. The synthesized NFs were reacted with lucigenin or luminol, and the CL signal obtained was integrated for 5 min. The integrated CL signal intensity was plotted against the metal type used for NF synthesis. As shown in Figure 2, lucigenin yielded the highest fluorescence intensity with Cu(I)-PEI-LA, which was 2.2 times higher than that of the polymer (PEI-LA). When lucigenin was replaced with luminol, PEI-LA did not produce CL signals. In contrast, NFs of PEI-LA with Cu(I), Cu(II), and Co(II) showed CL, and the most intense, integrated CL intensity was obtained from Cu(I)-PEI-LA-Ab NFs. As shown in Figure 2, Cu(I)-PEI-LA-Ab NFs produced the highest CL using either lucigenin or luminol; however, luminol produced 186 times stronger CL than lucigenin. Consequently, Cu(I)-PEI-LA-Ab NFs were the label of choice, and luminol was the CL reagent of choice for this study.

The difference in the CL responses of the six metal NFs (NFs: Cu(I), Cu(II), Fe(II), Co(II), Mn(IV), and Cd(II)) with lucigenin versus luminol arises from the ROS selectivity of each CL probe and the distinct catalytic profiles of the metals. Lucigenin is highly selective for superoxide anion radical (O_2_^·−^), reacting with its reduced radical form to produce light, whereas luminol responds to multiple ROS, including H_2_O_2_, ^−·^OH, and O_2_^·−^ (via one-electron oxidation) [25]. Different transition metals catalyze distinct ROS generation pathways from dissolved O_2_. Cu(I) NFs excel with both probes due to efficient Fenton-like cycling (O_2_ + Cu(I) → (Cu(II) + H_2_O_2_) → ^−·^OH), generating multiple ROS types, but particularly strong with luminol via the dominantly formed hydroxide anion radical, and it is less reactive with lucigenin as the main formed ROS are not superoxide (O_2_^·−^).

**Scheme 1:**
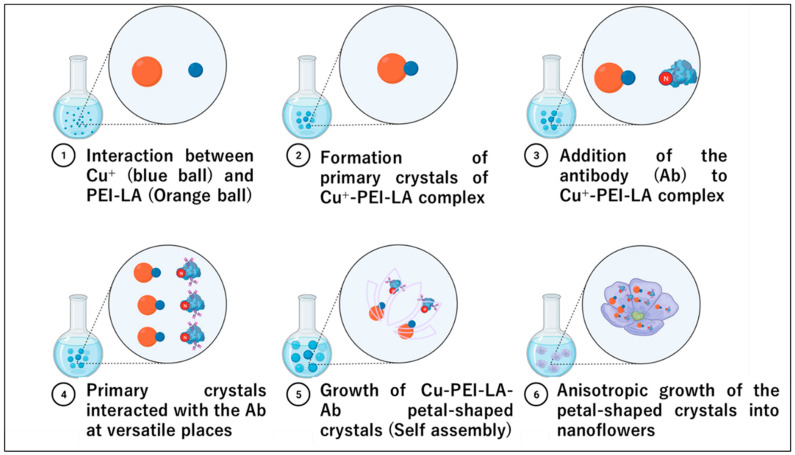
Steps in the formation of Cu(I)-PEI-LA Ab NFs.

During the “anisotropic growth” process, Ab is implanted or adsorbed into the outer layer of the petal when metal crystals with a petal form begin to self-assemble [26,27]. Finally, a hybrid NF was formed, as shown in Scheme 1.

### 3.3. Characterization of Cu(I)-PEI-LA-Ab NFs

**Figure 3:**
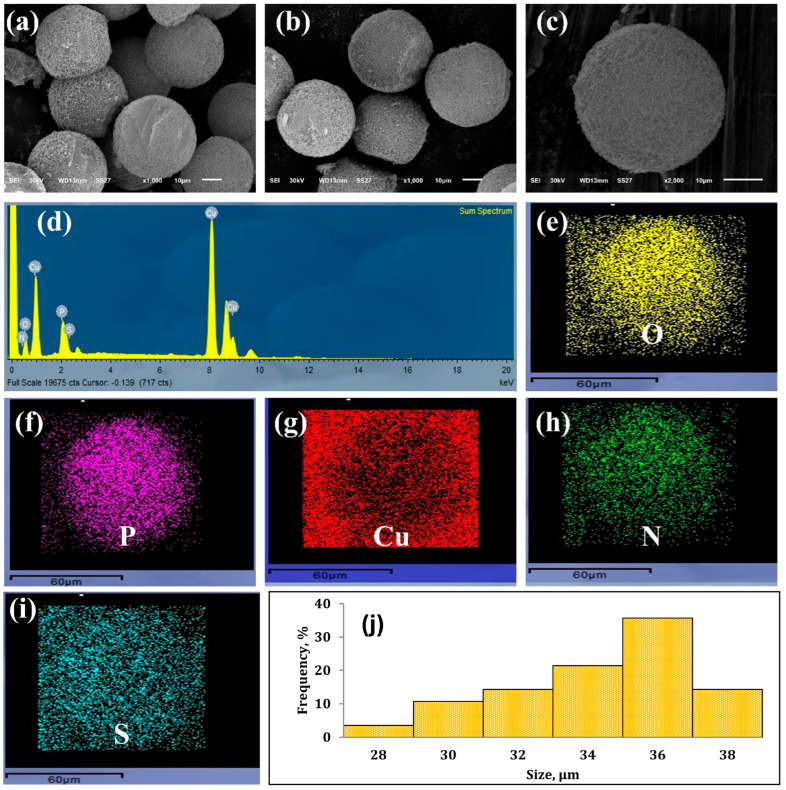
Characterization of Cu(I)-PEI-LA-Ab NFs: (**a**–**c**) SEM images. (**d**) EDX spectrum of sample. (**e**–**i**) Elemental mapping analysis of O, P, Cu, N, and S, respectively. (**j**) Size distribution curve of the NFs.

The nanostructure shape of the Cu(I)-PEI-LA Ab NFs was observed using SEM. The results are illustrated in Figure 3a–c, a round flower-like structure. According to the energy dispersive X-ray spectroscopy (EDX) and elemental mapping analysis (Figure 3d–i), Cu, N, O, S, and P were present within the Cu(I)-PEI-LA Ab NFs, confirming its elemental structure. Following the analysis of the SEM images using ImageJ software (1.52 a), the size of the NFs was determined to be 34.4 ± 3.6 μm (Figure 3j). This value is within the usual range of NF sizes described in the literature, which ranges from 2.0 to 35.0 μm [5,28,29].

**Figure 4:**
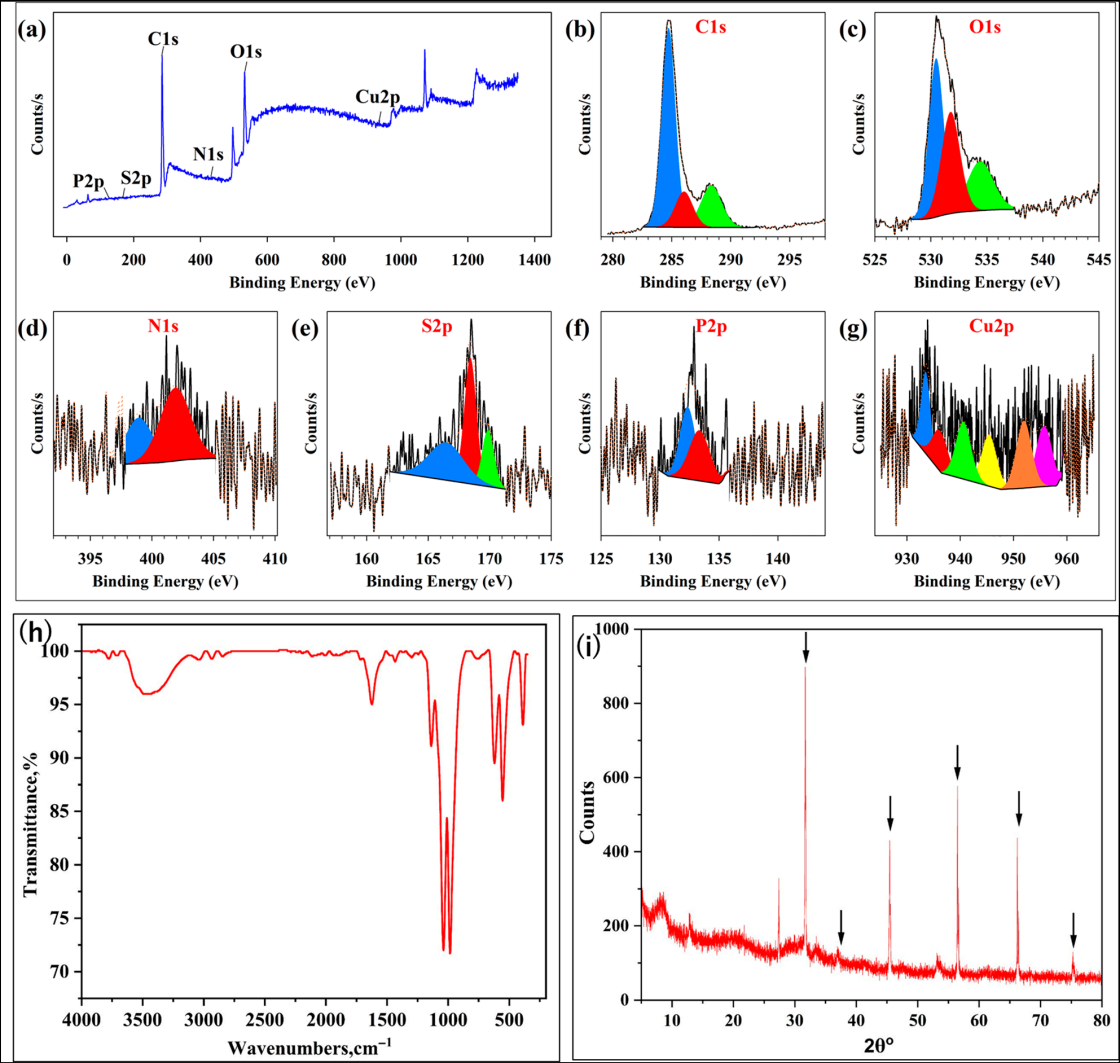
XPS, ATR-FTIR, and XRD characterization of the designed Cu(I)-PEI-LA-NFs, where XPS scans are (**a**) survey scan, (**b**) C1s scan, (**c**) O1s scan, (**d**) N1s scan, (**e**) S2p scan, (**f**) P2p scan, and (**g**) Cu2p scan, while (**h**) and (**i**) are ATR-FTIR and XRD spectra, respectively.

XPS analysis was performed to obtain further knowledge about the chemical states of the components included inside the Cu(I)-PEI-LA-NFs (Figure 4). The Cu(I)-PEI-LA-NF spectra displayed six peaks at 285.38, 532.16, 403.05, 168.48, 126.58, and 935.08 eV, which correspond to C1s, O1s, N1s, S2p, P2p, and Cu2p, respectively (Figure 4a). These peaks were observed in the region of the spectrum. The deconvolution of the XPS spectrum of C1s resulted in the appearance of three peaks at specific energies of 284.74, 286.13, and 288.36 eV. These peaks correspond to the C-C/C-S, C-O-C, and C=O functionalities, respectively (Figure 4b). The distinctive peaks of O-C, O-Cu, and O-P can be ascribed to the three peaks resolved in the O1s XPS spectra, located at 530.47, 531.77, and 534.41 eV, respectively (Figure 4c). The high-resolution N1s XPS spectrum was deconvoluted into two primary peaks at 398.94 and 401.91 eV, which are compatible with the distinctive peaks of H_2_N-C and N-H, respectively [30]. These peaks are shown in Figure 4d. The S2p peak was deconvoluted into three different types of peaks at 166.12 and 168.52 eV (Figure 4e) due to S-O/O-S-O, which is assigned to oxidized sulfur functionalities such as sulfate or sulfonate groups [31]. In conjunction with the creation of P=O and P-O bonds, which correspond to the peaks at 132.76 and 133.28 eV in the P2p spectrum (Figure 4f), the synthesis of Cu(I) phosphate was demonstrated [32]. During the deconvolution of the Cu2p spectra, two primary peaks (933.56 and 951.96 eV) and four satellite peaks (936.20, 940.62, and 945.44 eV) were observed (Figure 4g). The spin–orbit splitting of monovalent Cu(I) was responsible for the peaks at 933.56 and 951.96 eV. These peaks were attributed to the Cu2p3/2 and Cu2p1/2 peaks, respectively [33]. In addition, a peak at 955.71 eV was observed owing to the spin-orbit splitting of divalent Cu(II), which demonstrates that part of Cu(I) was oxidized to Cu(II) during the formation of the NFs [34].

Additionally, ATR-FTIR analysis was performed on the synthesized NFs to further characterize the surface functional groups and their coordination, and the results are shown in Figure 4h. The ATR–FTIR results were in good agreement with the XPS analysis and provided complementary evidence for the chemical interactions occurring in the Cu(I)–PEI–LA NFs. The ATR-FTIR spectrum of the Cu(I)–PEI–LA NFs verified the successful synthesis of the hybrid nanostructure via covalent bonding and metal coordination. The broad absorption band in the 3400–3300 cm^−1^ range is due to the N–H stretching vibrations of PEI [35,36]. This indicates that there is a significant amount of hydrogen bonding in the NF framework. The sharp bands at 1715 cm^−1^ and 1640 cm^−1^ correspond to the C=O stretching vibrations of the amide groups formed between LA and PEI [35,37]. The band at 1715 cm^−1^ is shifted from the expected position of amide C=O (1630–1690 cm^−1^), possibly due to metal coordination [38,39], possibly with Cu(I). These peaks indicate that amide bonds were formed between LA and PEI, and most of them were coordinated with Cu(I). The 1420 cm^−1^ corresponds to the bending vibrations of CH_2_ in the PEI backbone and possibly the symmetric stretching of carboxylate groups [40,41]. Further absorption in the 1100–1020 cm^−1^ range arises from the stretching vibrations of C–N and C–O, which further encourages the formation of amide bonds [36,37]. Additional bands in the low-frequency range of 620–550 cm^−1^ were assigned to Cu–S and Cu–N coordination vibrations. This confirms that Cu(I) ions were integrated into the NFs via chelation with the sulfur atoms of LA and the amine groups of PEI [41,42]. These spectral characteristics indicate that Cu(I)–PEI–LA NFs were successfully synthesized and held together by covalent amide bonds and Cu(I) coordination interactions.

Moreover, XRD analysis was performed on the synthesized NFs to characterize their crystalline structure and confirm the formation of Cu(1) NFs, and the results are shown in Figure 4. The XRD pattern of the PEI–LA–Cu nanoflowers shows multiple sharp reflections at 2θ = 31.69°, 36.90°, 45.46°, 53.15°, 56.48°, 66.22°, and 75.28°, using Cu Kα radiation, which collectively match the characteristic fingerprint of cubic cuprous oxide (Cu_2_O, cuprite phase), at ≈29.5–29.6° (110), 36.4° (111), 42.3° (200), 61.3° (220), and 73.5° (311) [43,44]. The presence of several sharp, intense peaks in the 30–80° range in the NF pattern, together with their approximate alignment with these standard Cu_2_O reflections, supports indexing the crystalline phase as cubic Cu_2_O with some peak position shifts and broadening as reported for Cu_2_O nanostructures and nanocomposites owing to strains, particle size, and organic coordination environment [44,45,46]. The absence of any distinct diffraction maximum at 2θ ≈ 43.3°, 50.4°, and 74.1°, where metallic copper (Cu^0^, fcc) exhibits its strongest (111), (200), and (220) reflections, rules out the presence of crystalline Cu^0^ in the nanoflowers [47,48] and indicates that copper is mainly stabilized in the Cu(I) state as Cu_2_O. In addition, no clear peaks are detected at ≈35.5° and 38.7°, which are the most intense reflections of monoclinic CuO, demonstrating that Cu(II) species do not form a significant crystalline CuO phase [43,49]. The coexistence of intense, well-resolved Cu_2_O peaks with a slightly elevated amorphous background is consistent with an organic–inorganic hybrid architecture in which crystalline Cu(I) domains of Cu_2_O are nucleated and stabilized within a PEI–lipoic acid matrix, as commonly observed for Cu_2_O–polymer and Cu_2_O–ligand hybrid systems [46,50]. Collectively, these features provide strong structural evidence that the synthesized nanoflowers consist of well-crystallized Cu(I) (Cu_2_O) domains integrated in a PEI–LA framework, confirming the successful formation of Cu(I)–PEI–LA nanoflowers suitable for antibody immobilization.

**Figure 5:**
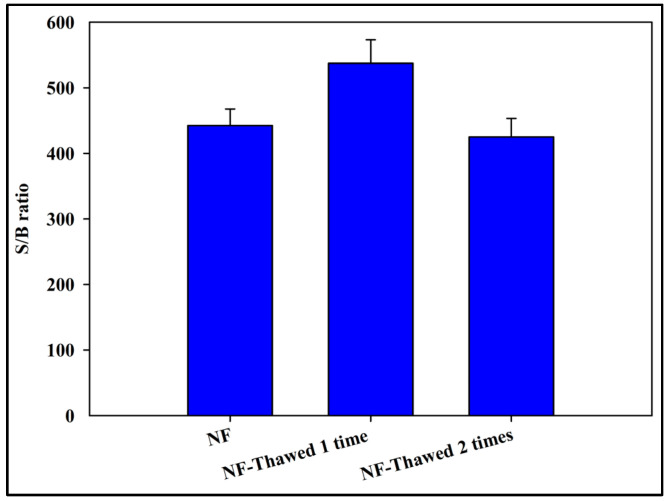
Effect of freeze–thaw cycles on the stability of the synthesized NFs.

The storage stability of the synthesized Cu(I)-PEI-LA NFs under freeze-thaw treatment was investigated. The Cu(I)-PEI-LA NFs were frozen at −20 °C for 2 days, then thawed at room temperature 1–2 times, and the CL generated by the reaction with luminol was measured. As shown in Figure 5, no significant change in the signal-to-blank (S/B) ratio was observed after two freeze-thaw cycles. Therefore, the activity of the synthesized Cu(I)-PEI-LA NFs was confirmed to be stable against freeze–thaw treatment.

### 3.4. Study of the CL Mechanism of the NFs

**Figure 6:**
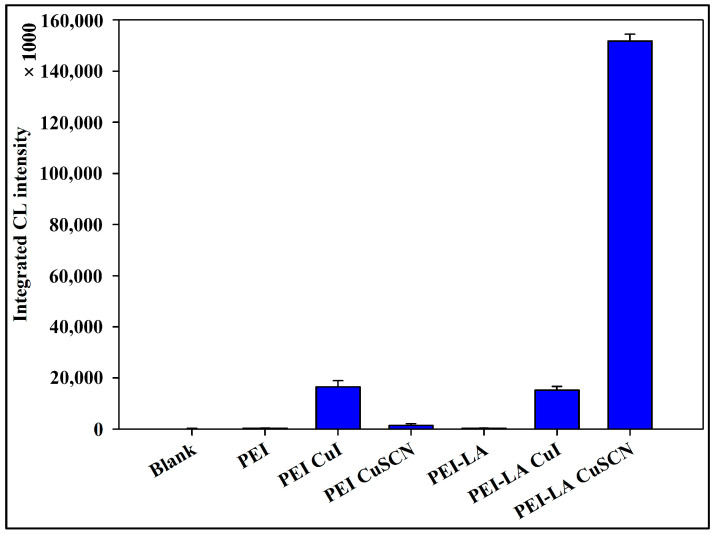
Elucidation of the structure–CL response relationship of Cu(I)-PEI-LA NFs.

As mentioned previously, our synthesized NFs could produce strong CL upon reaction with luminol in the absence of H_2_O_2_. To elucidate the relationship between the luminol CL response and the NF skeleton, different parts and types of NFs were prepared, and their CL responses to luminol were recorded. The tested substances included PEI, PEI-LA, PEI-CuI NFs, PEI-LA-CuI NFs, and PEI-LA-CuSCN NFs. The results are shown in Figure 6. Only NFs produced CL with luminol, while PEI and PEI-LA did not produce any significant CL response, demonstrating the necessity of the NF structure and metal (Cu(I)) for producing a CL response. Next, Cu(I)-PEI NFs showed a similar CL response when combined with PEI or PEI-LA. In contrast, a very strong CL response was observed only when Cu(I)-PEI-LA NFs were synthesized using Cu-SCN, which was then the NFs of choice for further applications as a signal multiplication immunoassay labeling tag for the detection of SARS-CoV-2 NP antigen.

Next, we aimed to elucidate the CL reaction mechanism of Cu(I)-PEI-LA. All luminol CL relations involve the formation of reactive oxygen species (ROS). We investigated the CL reaction between Cu(I)-PEI-LA Ab NFs and luminol in the presence of different ROS scavengers to determine which ROS contributed most to the CL reaction. The scavengers studied included catalase, sodium azide, SOD, and mannitol. The results of this study are summarized in Table 1. Among the tested ROS scavengers, SOD and sodium azide did not disrupt CL intensity; hence, it can be concluded that superoxide anion radicals and singlet oxygen have no role in the CL generation. In contrast, mannitol and catalase led to a significant decrease in CL intensity, indicating that hydroxide radicals and H_2_O_2_ are produced during NF CL generation. Next, the CL experiment was conducted after N_2_-purging to remove dissolved oxygen. This led to a very significant decrease in the CL of the NFs (Table 1). These complementary N_2_-purging data provide convergent evidence for the crucial role of dissolved oxygen in our developed CL assay system, which is also in accordance with the literature of luminol systems that use dissolved O_2_ as the main oxidant [51,52].

This mechanism, confirmed through ROS scavenging assays, relies on an in situ Fenton reaction. First, the dissolved oxygen is reduced by Cu(I) to form H_2_O_2_. The Cu(II) and H_2_O_2_ generated then undergo an in situ Fenton reaction, producing hydroxyl radicals that oxidize luminol, generating strong CL.

PEI provides a multidentate polycationic scaffold that coordinates Cu ions and creates a favorable microenvironment for Cu(I)/Cu(II) redox cycling, which likely facilitates charge transfer within the NFs [53,54,55]. LA units act as disulfide-containing linkers that anchor to Cu and may participate in redox-responsive S–S/S–H transformations, a behavior widely reported for LA-based materials [56,57,58,59], thereby contributing to the overall redox activity of the NFs. Taken together with the literature on PEI-based redox polymers and LA/dihydrolipoic acid redox couples, these results suggest that PEI assists electron transfer around the Cu centers and that LA contributes redox-responsive disulfide functionality in the NFs, creating an architecture that effectively cycles Cu(I)/Cu(II) species. Cu(I)PEI–LA–Ab NF tags provide catalytic robustness, high luminescence, and enhanced antigen-binding capability in a single nanostructure. In situ generation of H_2_O_2_ eliminates the use of external peroxide reagents, which helps stabilize the system and reduces breakdown variability in enzyme-based HRP/H_2_O_2_ systems. The new CL reaction demonstrates a second-generation H_2_O_2_-independent CL immunosensor powered by a Cu(I)-NF-based in situ Fenton reaction, paving the way for more robust, stable, and sustainable diagnostic systems in the future.

### 3.5. Application for the Determination of the Antigens Fixed on a Microplate Well

**Figure 7:**
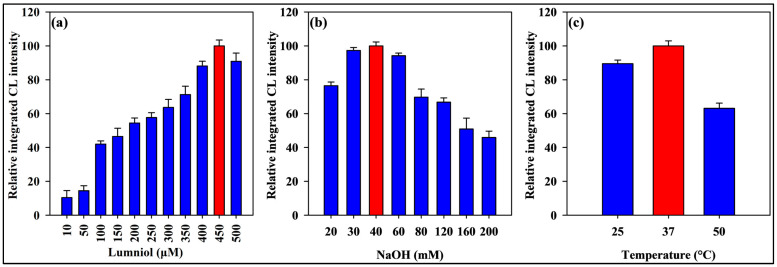
Optimization of the CL reaction condition between the Cu(I)-PEI-LA Ab NFs and luminol, showing the effect of (**a**) luminol concentration, (**b**) NaOH concentration, and (**c**) temperature on the relative integrated CL intensity. The relative integrated CL intensity is calculated as 100% for the optimum conditions in each case.

The synthesized Cu(I)-PEI-LA Ab NFs were used to determine the microplate-fixed SARS-CoV-2 nucleocapsid protein. The optimal conditions for CL measurement were established by experimenting with luminol concentrations ranging from 10.0 to 500.0 μm. Figure 7a shows that a luminol concentration of 450 μM yielded the best results. Additionally, the NaOH concentration was studied in the range of 20.0 to 200.0 mM, and the optimum concentration was found to be 40.0 mM Figure 7b. Furthermore, the external H_2_O_2_-free Fenton CL system reaction temperature was studied at 25, 37, and 50 °C (Figure 7c) using the microplate reader temperature control system. The optimum reaction temperature, which yielded the best CL response, was found to be 37 °C.

The selected alkaline pH (≈12.6, 40 mM NaOH) provides maximal CL efficiency consistent with established luminol–Fenton mechanisms. At this pH, luminol predominantly exists as its resonance-stabilized dianion (pKa ≈ 6.7 and 13.3), which undergoes efficient oxidation by hydroxyl radicals while maintaining Cu(I)/Cu(II) redox cycling. Lower pH values (<9) suppress luminol deprotonation, whereas excessively high alkalinity (>13) may destabilize Cu(I) species or induce hydroxide precipitation [60,61].

Regarding temperature, the CL intensity increases with temperature up to an optimum range of 25–40 °C, following Arrhenius kinetics for hydroxyl radical generation and luminol oxidation. Elevated temperatures (≥50 °C) can lead to Cu(I) oxidation to inactive Cu_2_O or thermal degradation of luminol. The selected temperature of 37 °C thus represents a practical excellence in terms of signal intensity, catalyst stability, and biological assay compatibility [60].

**Figure 8:**
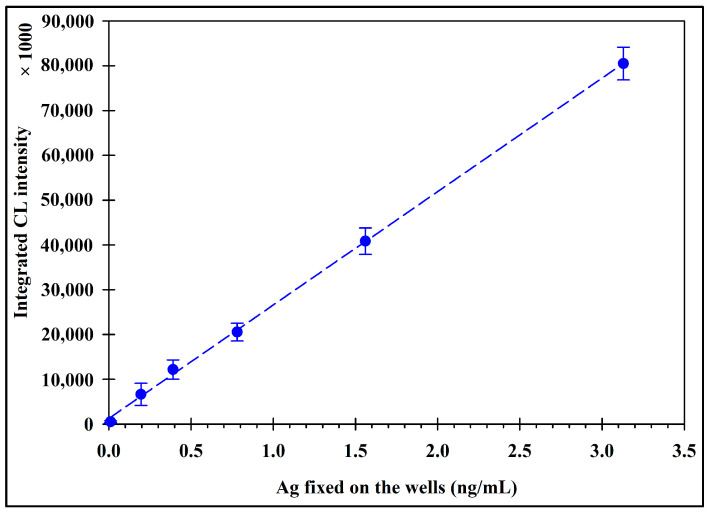
Calibration curve of SARS-CoV-2 fixed in wells.

Next, a calibration curve for the SARS-CoV-2 nucleocapsid protein solution, based on the CL generated by the Cu(I)-PEI-LA Ab NFs, was constructed (Figure 8). As summarized in Table 2, a good linear relationship was observed between the SARS-CoV-2 nucleocapsid protein concentration and the integrated CL intensity in the range of 0.01–3.13 ng/mL, with a determination coefficient (r^2^) of 0.9994. The limit of detection (LOD) for the SARS-CoV-2 nucleocapsid protein was 0.003 ng/mL (3SD/slope). Additionally, the recovery of the SARS-CoV-2 nucleocapsid protein Ag fixed in the wells was determined (Table 3). Excellent recoveries were obtained in the range of 97.7–109.2%, demonstrating the good reliability of the developed method for the detection of trace amounts of SARS-CoV-2 nucleocapsid protein Ag. In contrast to the widely used ELISA systems (luminol/H_2_O_2_ or TMB/H_2_O_2_), which are notoriously hazardous, irritant, and can cause acute toxicity, the luminol CL method has lower toxicity because it does not employ these reagents.

### 3.6. Application for the Determination of the Antigens Present in Samples Using a Competitive Immunoassay

**Figure 9:**
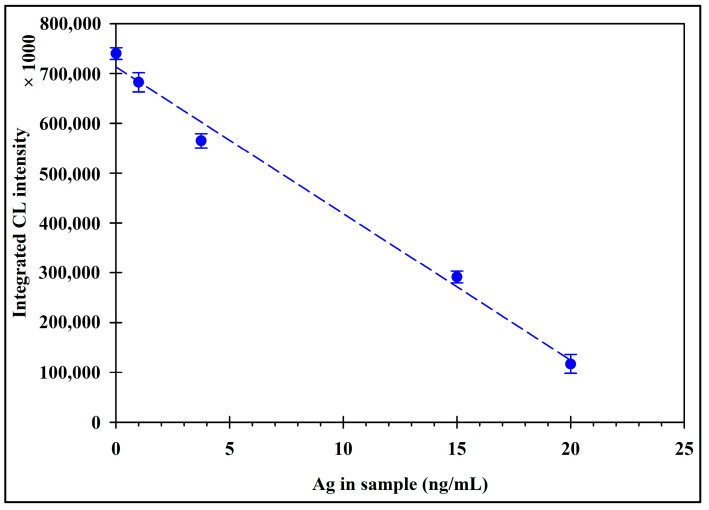
Calibration curve of SARS-CoV-2 in samples using a competitive immunoassay.

The newly developed NFs were applied for a non-enzymatic competitive immunoassay of SARS-CoV-2 nucleocapsid protein Ag using the procedure discussed in Section 2.6. Upon increasing the concentration of the tested SARS-CoV-2 nucleocapsid protein antigen, it competed with the well-fixed antigen for the NFs, and the CL decreased gradually in a concentration-dependent manner. The calibration curve for the SARS-CoV-2 nucleocapsid protein solution using the competitive immunoassay is shown in Figure 9. As shown in Table 4, a good linear relationship was observed between the SARS-CoV-2 nucleocapsid protein concentration and the integrated CL intensity in the range of 0.1–20 ng/mL, with a determination coefficient (r^2^) of 0.9903. The detection limit (3SD/slope) for SARS-CoV-2 nucleocapsid protein was 0.01 ng/mL. Additionally, the recovery of the SARS-CoV-2 nucleocapsid protein Ag fixed in the wells was determined (Table 5). Good recoveries were obtained in the range of 93.63 to 107.1%, demonstrating the very good reliability of the developed method for the detection of trace amounts of SARS-CoV-2 nucleocapsid protein Ag in samples.

**Figure 10:**
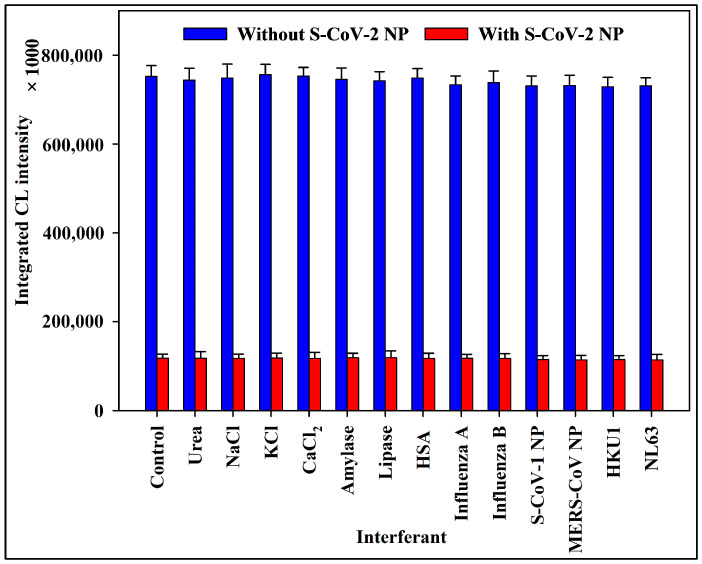
Selectivity of the developed NF-based competitive immunoassay for SARS-CoV-2 in the presence of interfering salts, proteins, influenza viruses, and other common coronaviruses. The concentration of NP used was 15.0 ng/mL, whereas the concentration of enzymes and salts was 1000.0 ng/mL.

It is important to highlight that our competitive NF-based immunoassay is naturally selective for SARS-CoV-2 NP because we incorporated SARS-CoV-2 NP detection antibodies into the NF structure. We verified that our SARS-CoV-2 competitive NF-based immunoassay was selective for influenza A and B viruses and common human coronaviruses. We also examined other potential interferences, including salts and enzymes that might be present in the samples. There was hardly any reaction to the interference in the experiment. Figure 10 further shows that these interferents did not significantly alter the signal when combined with SARS-CoV-2 NP.

The analytical performance of our NF-based immunoassay was compared with that of a commercially available sandwich ELISA. ELISA quantifies SARS-CoV-2 NP by measuring the chromogenic signal (from TMB) of a streptavidin–HRP immunocomplex. Statistical analysis using Student’s *t*-test and an F-test revealed no significant difference between the two methods (Table 5) in terms of accuracy (*p* = 0.91) and precision (*p* = 0.33). Despite this comparable performance, our proposed NF-based competitive immunoassay method exhibited a significantly lower LOD. With an LOD of 0.01 ng/mL, 38 times lower than that of ELISA (0.38 ng/mL), our method demonstrated a substantial increase in sensitivity.

Finally, our novel labeling approach was compared with previous enzymatic and non-enzymatic organic–inorganic nanohybrids, including CL [5,62], electrochemical [63,64,65,66], impedimetric [67], fluorogenic [4,68], and chromogenic [69,70,71] tags used for detecting SARS-CoV-2, as shown in Table 6. The developed Cu(I)-PEI-LA demonstrates superior performance, with a lower LOD than the other methods’ sensitivity (LOD) using simple luminol as the CL reagent in the absence of H_2_O_2_. Being the first of its kind, our organic–inorganic hybrid NFs utilize the redox properties of the LA-functionalized PEI backbone polymer and the catalytic power of Cu(I) to catalyze the generation of Fenton’s reaction-induced CL signals in immunoassays without the need for H_2_O_2_. Additionally, the analysis time of the proposed method per sample was also compared with previously reported approaches, revealing comparable or up to 100-fold faster analysis in some cases. This marked improvement arises from the use of microplate-based assays, which enable the simultaneous processing of up to 96 samples under identical conditions, thereby dramatically increasing analytical throughput.

## 4. Conclusions and Future Perspectives

In conclusion, this study successfully developed a novel H_2_O_2_-free CL immunoassay for ultrasensitive detection of SARS-CoV-2 NP. The core innovation lies in the design of Cu(I)-PEI-LA-Ab NFs, which functions as a robust non-enzymatic label. This NFs tag drives a Fenton-like reaction, in which Cu(I) reduces the dissolved oxygen to generate hydroxyl radicals in situ, which subsequently oxidize luminol to produce an intense and sustained CL signal. This mechanism eliminates the dependency on unstable exogenous H_2_O_2_, a major bottleneck in conventional HRP-based assays. The Cu(I)-PEI-LA-Ab NFs demonstrated superior CL performance, outperforming other metal-based NFs by over 100-fold. The developed immunoassay platform exhibited excellent analytical performance, with a wide linear range (0.01–3.13 ng/mL), sub-picomolar limit of detection (0.003 ng/mL), and high accuracy in both direct and competitive assay formats. The successful implementation of a competitive immunoassay format demonstrates its direct applicability in the analysis of complex real-world samples. In this format, the decreasing CL signal reliably correlated with increasing antigen concentrations in solution, and the method yielded excellent recovery rates (90.7–113.1%), confirming its robustness and accuracy, even in the presence of potential matrix interferents. The stability, simplicity, and high sensitivity of the system establish it as a powerful and reliable tool for clinical diagnostics, paving the way for more robust and sustainable bioanalytical platforms.

The potential of this peroxide-free CL system extends far beyond SARS-CoV-2 detection. Its robustness and high signal output make it an ideal drop-in replacement for enzymatic labels such as HRP in a wide range of existing immunoassay formats. The stability of the NFs label and the simplicity of the “add-and-measure” CL reaction render this system an excellent candidate for integration into portable, low-cost point-of-care devices. This could facilitate rapid testing in clinics, pharmacies, and even at home. In addition, the proposed principle is universally applicable. By simply changing the conjugated Ab, the assay can be reconfigured for the detection of various proteins, such as cancer biomarkers (e.g., prostate-specific antigen (PSA) and carcinoembryonic antigen (CEA)), cardiac markers (e.g., troponin), or hormones, significantly impacting early disease diagnosis and monitoring. Moreover, the system can be deployed to detect small-molecule contaminants (e.g., pesticides and toxins) using a competitive immunoassay format or to monitor bacterial pathogens (e.g., *E. coli* and *Salmonella*) in water and food samples.

## Figures and Tables

**Table 1:** ROS scavenger reagents, ROS type, and corresponding CL decrease % in Cu(I) PEI-LA-Ab NFs.

ROS/Oxygen Scavenger Reagent	Type of Removed ROS	CL Decrease Percentage
Catalase (10 U/mL)	H_2_O_2_	↓ 25%
Catalase (100 U/mL)		↓ 95%
Sodium azide (NaN_3_) (0.02 mM and 1.0 mM)	Singlet oxygen ^1^O_2_ or metallic nanozyme inhibitor	No significant decrease
SOD (10 and 100 U/mL)	Superoxide radical O_2_^·−^	No significant decrease
Mannitol (0.02 mM)	Hydroxyl radical ^−·^OH	↓ 37%
Mannitol (25.0 mM)		↓ 99.2%
Nitrogen purging	Dissolved oxygen	↓ 97.5%

**Table 2:** Calibration data and LOD of SARS-CoV-2 nucleocapsid protein Ag fixed on the wells.

Linear Range	Calibration Equation	r^2^	LOD
0.01–3.13 ng/mL	Y = 25.34 × 10^6^ X + 1.24 × 10^6^	0.9994	0.003

Y = CL intensity. X = SARS-CoV-2 nucleocapsid protein concentration (ng/mL).

**Table 3:** Recovery of SARS-CoV-2 nucleocapsid protein Ag fixed on the wells.

Concentration Fixed on the Well (ng/mL)	Concentration Found	Recovery %
0.195	0.209	107.18
0.39	0.426	109.23
0.78	0.762	97.69
1.56	1.57	100.64
3.13	3.12	99.68

**Table 4:** Calibration data and LOD of SARS-CoV-2 nucleocapsid protein Ag using the NF-based competitive immunoassay.

Linear Range	Calibration Equation	r^2^	LOD
0.10–20.0 ng/mL	Y = −29.49 × 10^6^ X + 714.16 × 10^6^	0.9903	0.01

Y = CL intensity. X = SARS-CoV-2 nucleocapsid protein concentration (ng/mL).

**Table 5:** Recovery of the SARS-CoV-2 nucleocapsid protein Ag using the NF-based competitive immunoassay.

Developed NF Method			Reference ELISA Method	
Concentration Added (ng/mL)	Recovery (%)	Precision (RSD, %)	Concentration Added (ng/mL)	Recovery (%)
0.1	0.1040	104.0	0.39	97.40
1	0.9967	99.67	1.56	107.7
3.8	3.558	93.63	3.125	98.37
15	16.07	107.1	6.25	95.72
20	18.81	94.05	12.5	101.3
Student’s *t*-test *p*-value			0.9073	
Variance ratio F-test *p*-value			0.3296	

**Table 6:** Comparison between the created Cu(I)-PEI-LA label and the reported labels used to identify SARS-CoV-2-NP.

Label	Linear Range (ng/mL)	LOD (ng/mL)	Total Analysis Time (min)	Detection Method	Ref.
Cu(I)-PEI-LA Ab NFs for Ag fixed on the well	0.01–3.13	0.003	130 min/96 samples = 1.35 min/sample	CL	Developed label
Cu(I)-PEI-LA NFs for competitive immunoassay of Ag	0.1–20.0	0.01	130 min/96 samples = 1.35 min/sample	CL	Developed label
Co-Fe@hemin Nanozyme	0.2–100	0.1	15 min/sample	CL	[62]
Cu(II)-Chitosan-alizarin NFs	0.2–25	0.013	130 min/96 samples = 1.35 min/sample	CL	[5]
Au NPs modified electrode	1–10	2.6	140 min/sample	Electrochemical	[63]
Au modified electrode	500–5000	39.0	75 min/sample	Impedimetric	[64]
Ti/Au patterned electrodes	15.5–1,550,000	15.0	30 min/sample	Electrochemical	[65]
Carbon nanostructured	0.5–100	2.0	15 min/sample	Electrochemical	[66]
Reduced graphene oxide doped electrode	160–1250	150.0	60 min/sample	Impedimetric	[67]
Carbon dots	0.1–100,000	0.1	180 min/96 samples = 1.9 min/sample	Fluorogenic	[68]
Zn(II)-Chitosan-alizarin NFs + Phenylboronic acid	0.39–12.5	0.04	130 min/96 samples = 1.35 min/sample	Fluorogenic	[4]
Zn(II)-Chitosan-alizarin NFs + Boronic acid	0.52–3.13	0.52	130 min/96 samples = 1.35 min/sample	Fluorogenic	[4]
Oligonucleotide carrying Enzymes	5–5000	2.16	165 min/96 samples = 1.7 min/sample	Chromogenic	[69]
Au@Pt tag	0.1–100	0.01	80 min/sample	Chromogenic	[70]
Nanozyme-tagged immunochromatography	0.05–1.6	0.03	25 min/sample	Chromogenic	[71]

## Data Availability

The original contributions presented in this study are included in the article. Further inquiries can be directed to the corresponding authors.
